# Supplementary material for: Effects of Prone Positioning on Head Control in Preterm Infants: Randomized and Controlled Clinical Trial Protocol
Source: Int J Environ Res Public Health. 2023 Jan 29;20(3):2375. doi: 10.3390/ijerph20032375 (PMC9915948; doi:10.3390/ijerph20032375)
Supplement: Supplementary file 1 [file ijerph-20-02375-s001.zip › ijerph-2073221-supplementary.pdf]

WHAT IS TUMMY TIME?

It's the moment of the day that the baby plays laying on their stomachs when...

- Awake
- Under supervision

Start with short periods and increase with time!

Start with short periods of 2 – 3 minutes until you complete 30 minutes daily of Tummy Time.

Respect your baby’s limits by recognizing their signs oft iredness:

- Crying
- Resting head on surface

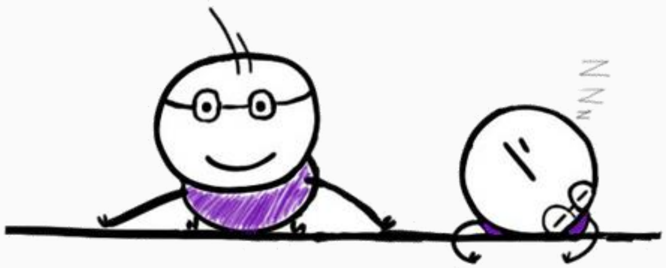

You can stop Tummy Time even before your baby gets tired

CONTACT US!

Department of Physical Therapy - Federal University of Rio Grande do Norte (UFRN)

gpsafeufrn@gmail.com
@gpsafeufrn

TUMMY TIME

How to do it with my baby?

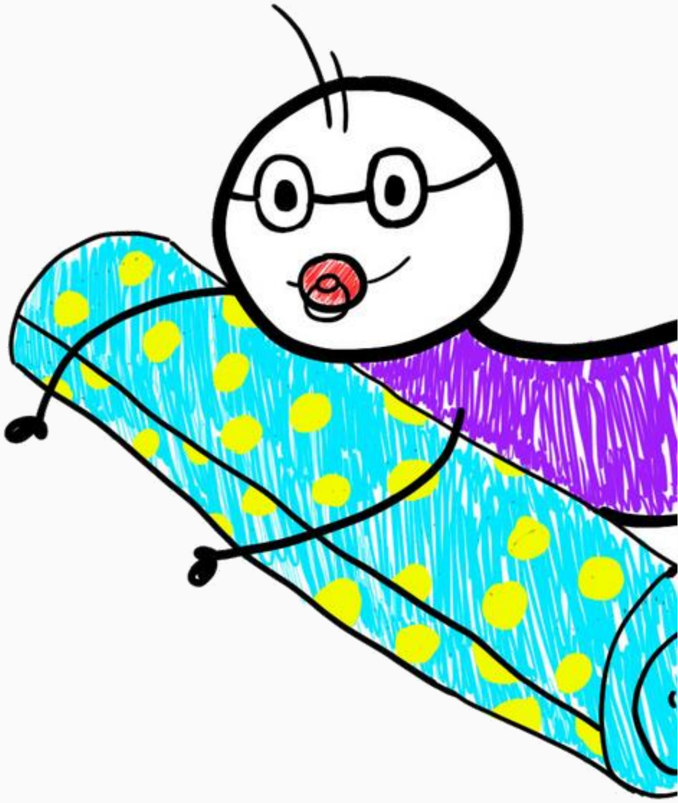

UFRN UNIVERSIDADE FEDERAL DO RIO GRANDE DO NORTE

WHY SHOULD I DO IT?

- It helps strengthen head, neck and upper boddy muscles
- Improves motor development and motor skills
- Prevents Plagiocephaly (flat spots of the back of the baby's head)

MAKE IT A DAILY ROUTINE

Insert Tummy Time into your usual daily activities:

- When drying the baby after shower
- When changing diapers or applying lotion
- Play time

After burping your baby, try placing him belly-down on your chest.

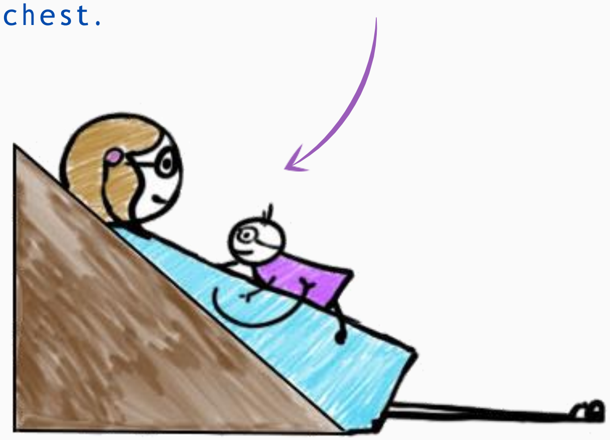

STIMULATE THE BABY

Stimulate your baby's ability to reach and play:

- Arrange toys in a circle around your baby, give preference to colorful toys that make sounds and arouse interest like a mirror

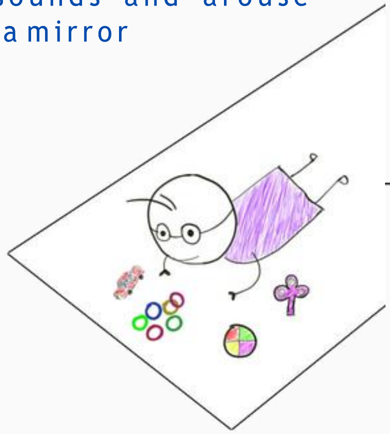

- Sit or lie down in front of the baby
- Hold a toy in front of the baby's face to get his attention

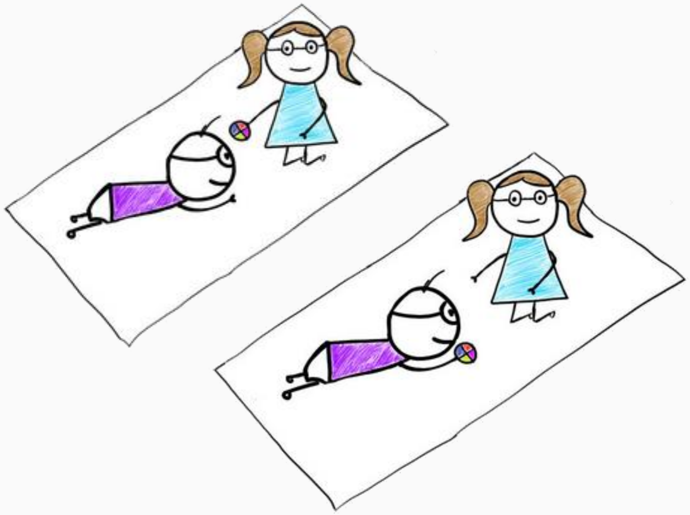

IMPROVE THE BOND

Invite your family to join Tummy Time

- Make eye contact, speak or sing
,

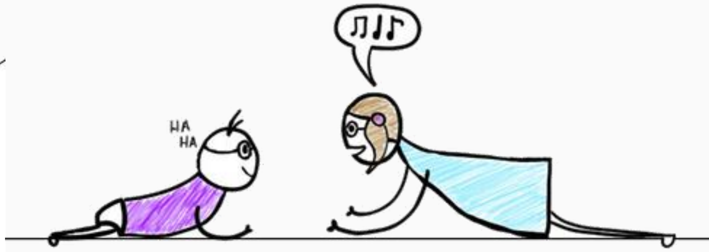

TIPS

Chest support

- Roll a thin towel or blanket and place it under your baby's chest and place his arms over the roll with his hands outstretched in front

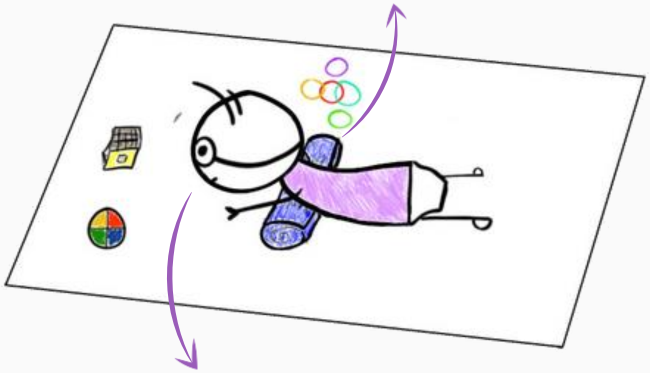

Your baby's chin should always be positioned in front of the chest support so that the nose and mouth are free
